# The genetic architecture of mitochondrial dysfunction in Parkinson’s disease

**DOI:** 10.1007/s00441-017-2768-8

**Published:** 2018-01-25

**Authors:** S. B. Larsen, Z. Hanss, R. Krüger

**Affiliations:** 1 Luxembourg Centre for Systems Biomedicine (LCSB), University of Luxembourg, Esch-sur-Alzette, Luxembourg; 2 Parkinson Research Clinic, Centre Hospitalier de Luxembourg (CHL), Luxembourg City, Luxembourg

**Keywords:** Parkinson’s disease, Mitochondria, Quality control, Risk factors, Genetics

## Abstract

Mitochondrial impairment is a well-established pathological pathway implicated in Parkinson’s disease (PD). Defects of the complex I of the mitochondrial respiratory chain have been found in post-mortem brains from sporadic PD patients. Furthermore, several disease-related genes are linked to mitochondrial pathways, such as *PRKN*, *PINK1*, *DJ-1* and *HTRA2* and are associated with mitochondrial impairment. This phenotype can be caused by the dysfunction of mitochondrial quality control machinery at different levels: molecular, organellar or cellular. Mitochondrial unfolded protein response represents the molecular level and implicates various chaperones and proteases. If the molecular level of quality control is not sufficient, the organellar level is required and involves mitophagy and mitochondrial-derived vesicles to sequester whole dysfunctional organelle or parts of it. Only when the impairment is too severe, does it lead to cell death via apoptosis, which defines the cellular level of quality control. Here, we review how currently known PD-linked genetic variants interfere with different levels of mitochondrial quality control. We discuss the graded risk concept of the most recently identified PARK loci (*PARK* 17–23) and some susceptibility variants in *GBA*, *LRRK2* and *SNCA*. Finally, the emerging concept of rare genetic variants in candidates genes for PD, such as *HSPA9*, *TRAP1* and *RHOT1*, complete the picture of the complex genetic architecture of PD that will direct future precision medicine approaches.

## Introduction

Patients with Parkinson’s disease (PD) experience motor impairments such as resting tremor, bradykinesia, rigidity and postural instability but also non-motor symptoms such as sleep perturbations, constipation, cognitive impairment or depression (Krüger et al. 2017). The diversity of symptoms indicates that, beyond the degeneration of dopaminergic neurons in the substantia nigra responsible for the typical movement disorder, other neuronal subtypes like cholinergic, serotonergic and noradrenergic neurons are also affected in the central and enteric nervous system (Krüger et al. 2017). However, the molecular mechanisms underlying this neuronal cell death are still not fully understood. The identification of rare monogenic forms of PD and subsequent functional studies related to disease-causing mutations have substantially advanced our understanding of the cellular dysfunction driving neurodegeneration during the last 20 years (Antony et al. 2013). Indeed, studying individual genetic mutations allows the definition of cellular phenotypes linked to impaired molecular signalling pathways related to a specific gene. By linking the cellular phenotypes discovered in patient-based models of monogenic forms of PD, common molecular patterns have emerged. These phenotypes may subsequently serve as cellular prototypes for typical sporadic form of the disease (Antony et al. 2013).

**Fig. 1:**
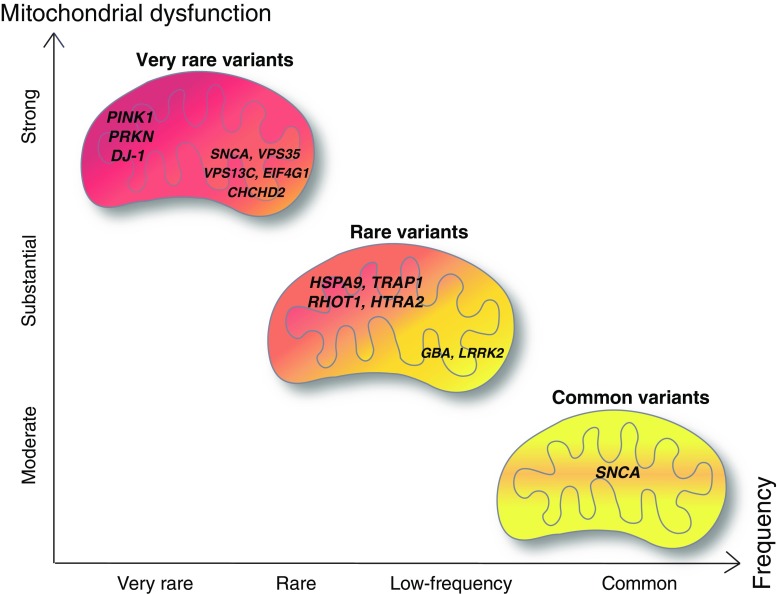
Graded risk concept for genes related to mitochondrial dysfunction in Parkinson disease. Severity of mitochondrial dysfunction is represented in a gradient from *red* (strong mitochondrial dysfunction) to *yellow* (moderate mitochondrial dysfunction). Figure adapted from Manolio et al. 2009

One pathway that is well established among all these disease-associated pathological pathways involves mitochondrial dysfunction as a shared feature between sporadic and monogenic PD. The finding of defects of specific complexes of the mitochondrial respiratory chain, i.e., complex I deficiency, in post-mortem brains from sporadic PD patients (Parker et al. 2008; Schapira et al. 1990) indicated an important role of mitochondria in the pathogenesis of the disease. These findings were recently substantiated by the identification of mutations in several PD-linked genes that have been shown to specifically induce mitochondrial impairment. Consistently across different models, mitochondrial dysfunction was observed for autosomal recessively inherited forms of PD caused by mutations in the genes coding for PINK1, Parkin and DJ-1 proteins (Valente et al. 2004; Lücking et al. 2000; Bonifati et al. 2003) (Fig. 1).

Based on linkage studies and next-generation sequencing technologies like whole-exome or whole-genome sequencing, more recently an increasing number of genes has been linked to monogenic PD, expanding the current total of *PARK* loci to 23 (Lill 2016). Moreover, common variants in *PARK* loci (e.g., polymorphisms in *SNCA*) or other disease-associated genes (e.g., *GBA*) arise as risk factors and relate hereditary to the more common sporadic form of PD (Schiesling et al. 2008; Sidransky et al. 2009). Studying pathological pathways in the frame of these PD-linked mutations further underscored the central role played by mitochondria at different levels of quality control and energy supply to ensure normal function of the cell. Here, we review how currently known PD-linked genetic variants may impact mitochondrial function due to interference with the different levels of mitochondrial quality control. In this context, we refer to the graded risk concept representing the complex genetic architecture of PD (Fig. 1) and focus on the most recently identified PARK loci (PARK 17–23) and susceptibility variants for sporadic PD (such as *GBA*, *LRRK2*) including common risk factors such as *SNCA* polymorphisms.

## Relevance of mitochondria for cellular homeostasis

Mitochondria are highly dynamic organelles that are essential to maintain cellular function. These organelles maintain neuronal function and integrity via sustained energy supply for important cellular functions including synaptic activity or calcium buffering after depolarisation (reviewed in Bingol and Sheng 2016). Consequently, a tight regulation of mitochondrial homeostasis especially in neurons is necessary to maintain cellular processes. For example, reactive oxygen species (ROS) play a role in mitochondrial signalling but if the concentration gets out of range, oxidative stress might arise and damage biological molecules and structures like DNA, proteins or lipid membranes (Bingol and Sheng 2016). The production of adenosine triphosphate (ATP) by oxidative phosphorylation in the mitochondrial electron transport chain (ETC) is one of the key mitochondrial functions to provide energy. This process is accompanied by the passage of protons in the inter-membrane space, which subsequently creates the mitochondrial membrane potential (MMP) (Fernie et al. 2004). In case of an imbalanced MMP, production of a high level of ROS such as superoxide may arise due to electron leakage. Thus, the mitochondrion has to ensure a balance to efficiently produce ATP without releasing a pathological level of ROS.

The MMP also regulates calcium (i.e., Ca2+) entry in the mitochondria via the mitochondrial Ca2+ uniporter. The balance between Ca2+ accumulation in mitochondria and release via mitochondrial Na+/Ca2+ and H+/Ca2+ exchangers allows mitochondria to have a high capacity of buffering cytosolic Ca2+ (Rizzuto et al. 2012). Particularly in neurons, the regulation of Ca2+ is necessary for neurotransmitter release, metabolism and cell survival. Mitochondria are creating tight contacts with the main Ca2+ stock in the cell, the endoplasmic reticulum (ER), in domains called mitochondrial associated membranes (MAM). Release of Ca2+ taken up by the mitochondria physiologically increases ATP production. However, Ca2+ overload may lead to mitochondrial membrane permeabilisation, followed by cytochrome c release and subsequent apoptotic cell death (Kroemer et al. 2007). The role of Ca2+ as an essential secondary messenger within the cell but also as a trigger of cell death, shows the importance of a well-balanced Ca2+ homeostasis.

**Fig. 2:**
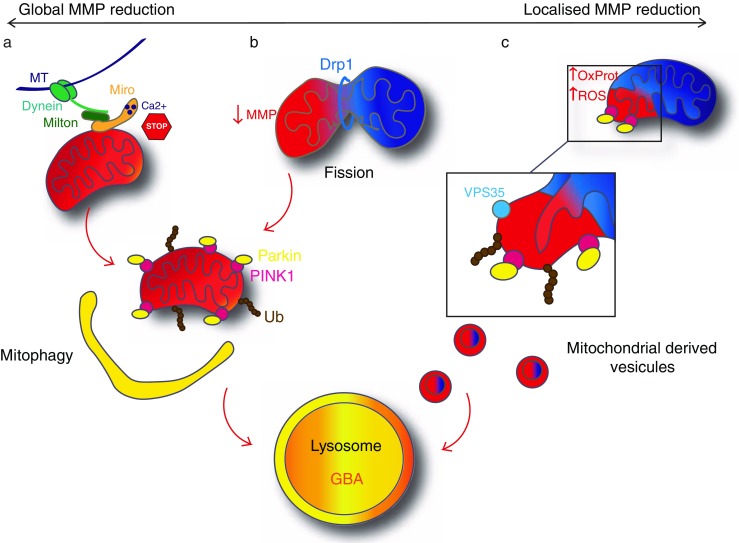
Organellar quality control. (**a**) Major mitochondrial dysfunction leads to a global MMP reduction (*red*). Miro1 ensures the arrest of mitochondrial transport along the microtubules (*MT*) by sensing the calcium concentration. PINK1 accumulates on the mitochondria and recruits Parkin. Parkin ubiquitinates various mitochondrial proteins that leads to the engulfment of mitochondria by the autophagosome. The autophagosome fuse with the lysosome and mitochondrial proteins become degraded by lysosomal enzymes. This process is called mitophagy. **b** Global mitochondrial dysfunction can be avoided by fission, induced by Drp1. The impaired daughter mitochondria (*red*) will then undergo mitophagy as well. **c** Mitochondria can also have a localised MMP reduction due to local increase of oxidised proteins (*OxProt*) or ROS. This leads to a budding of mitochondria-derived vesicles (MDVs) implicating a local activation of PINK1/Parkin pathway and recruitment of VPS35. MDVs then fuse with the lysosome. Healthy mitochondria (physiological MMP) are represented in *blue*, dysfunctional mitochondria (decreased MMP) are represented in *red*. Proteins in *bold* are linked to PD

Mitochondrial dynamics play an important role in the maintenance of organellar homeostasis (Burbulla and Krüger 2011). Particularly in neurons, mitochondria need to be very mobile to furnish ATP at sites of energy consumption but also to buffer Ca2+ necessary for neurotransmission at the synapses. Mitochondria can travel along microtubules via association to kinesin-1 via the Miro–Milton complex (reviewed in Cai and Sheng 2009). Another way for mitochondria to become distributed in the cell in response to local energy demand is undergoing fission. Fission and fusion events control mitochondrial morphology and need to be well balanced for the mitochondrial network to work properly. These events regulate respiration, calcium homeostasis, clearance and distribution of mitochondria (reviewed in Bingol and Sheng 2016). The fusion machinery involves three GTPases: Mfn1, Mfn2 and OPA1. Mfn1 and Mfn2 mediate outer-membrane fusion whereas OPA1 is implicated in inner-membrane fusion (Mishra and Chan 2016). Fusion of two mitochondria is involved in the quality control process. In neurons, the fusion rate is very important as mitochondrial damage can lead to the loss of respiratory chain activity and in the long term to neurodegeneration (Chen et al. 2007). Fusion events can cope with minor damage to mitochondrial DNA or slight disequilibrium of the homeostasis (reviewed in Twig and Shirihai 2011). Indeed, the fusion of two mitochondria allows the mix of their content that will dilute the damaged elements. If this phenomenon is not sufficient to cope with the damage, mitochondria can undergo fission. Fission defines the symmetric or asymmetric cleavage of a mitochondrion into two parts and is also implicated in quality control as it can be the first step towards the clearance of fragments of mitochondria via the lysosomal pathway (Fig. 2b). Fission enables to sequester damaged parts of the mitochondria. It requires the GTPase Drp1 (Dynamin-related protein 1) that is recruited to the mitochondrial outer membrane. During fission, mitochondria endures a drop of membrane potential. Drp1 is then forming a spiral around the mitochondria in order to perform the division into two daughter mitochondria (Fig. 2b). If the depolarisation goes below a certain level, these daughter mitochondria will go towards the mitophagy fate (Twig and Shirihai 2011).

**Fig. 3:**
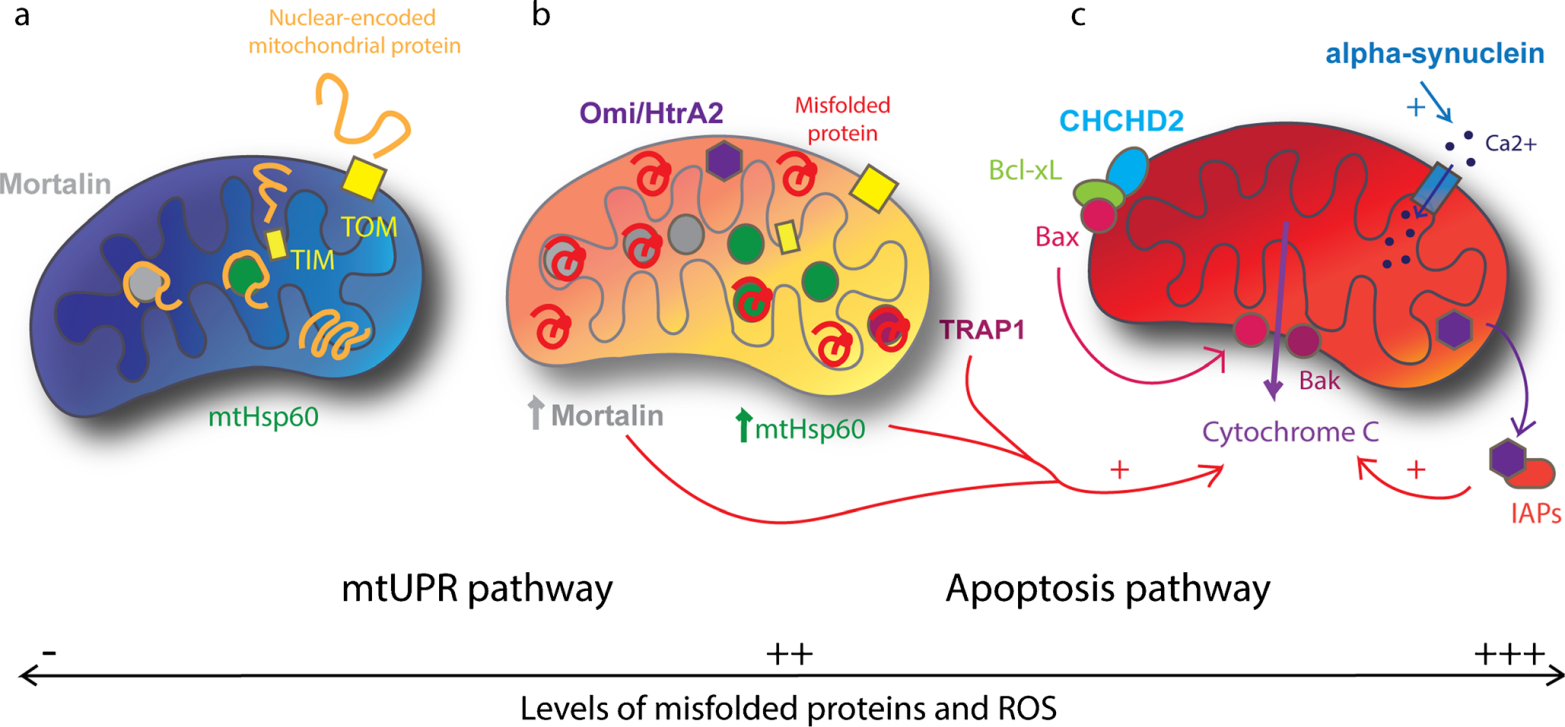
Molecular and cellular quality control. **a** In healthy mitochondria (*blue*), unfolded proteins from the cytoplasm enter by TOM and TIM. Inside the mitochondria, Mortalin and mtHsp60 help in the folding of proteins. **b** In case of increased level of misfolded proteins and ROS within the mitochondria (*orange*), the mtUPR pathway is activated. This leads to the increase of mitochondrial chaperones expression, Mortalin and mtHsp60, which help in the refolding of misfolded and oxidised proteins. TRAP1 and Omi/HtrA2 also help to refold or degrade the misfolded proteins. **c** In case of major cellular dysfunction, impaired mitochondria (*red*) triggers apoptosis. CHCHD2 allows relocalisation of Bax, which conjointly with Bak induces release of cytochrome c. TRAP1, mtHsp60 and mortalin can stimulate cytochrome c release and Omi/HtrA2 facilitates apoptosis by binding to IAPs. Also, alpha-synuclein increases Ca2+ transfer to the mitochondria that, when overloaded, induces apoptosis. Proteins in *bold* are linked to PD

Slight changes in mitochondrial homeostasis may have a substantial impact on organellar function and even on the integrity of the whole cell. For this reason, mitochondrial homeostasis is finely regulated and the mitochondria need an effective quality control machinery that can be subdivided into three levels: molecular, organellar and cellular quality control (Baker et al. 2011). When mitochondrial homeostasis is unbalanced, the molecular level is the first quality control step to be involved. It implicates the mitochondrial unfolded protein response (mtUPR) with proteases and chaperones such as mtHsp60 (mitochondrial Heat shock protein 60), TRAP1 (TNF receptor associated protein 1) and mortalin (HSPA9), which re-fold damaged proteins or ultimately cleave and clear them from the mitochondria (Burbulla and Krüger 2011) (Fig. 3a, b).

If the deregulation becomes more severe, an organellar quality control level is required to sequester damaged mitochondria in part or as a whole (Jin and Youle 2013) (Fig. 2). This involves mitochondrial-derived vesicles (MDVs) to eliminate larger amounts of misfolded proteins or excessive ROS (Soubannier et al. 2012, b; McLelland et al. 2014) (Fig. 2c) or mitophagy to eliminate greater fragments or even the whole organelle (Rakovic et al. 2013) (Fig. 2a, b). The PINK1/Parkin pathway is critically involved in both mechanisms (Sugiura et al. 2014; Narendra et al. 2008).

Finally, if mitochondrial homeostasis cannot be rescued, the permeability transition pore opens and leaks cytochrome c into the cytoplasm that activates the apoptosis pathway (Kroemer et al. 2007) (Fig. 3c). This defines the cellular level of quality control and prevents ultimate damage to the organism.

## Mitochondrial dysfunction caused by PD-related genes: the role of organellar quality control

Organellar quality control is critically related to the term “mitophagy”, which is defined as the selective lysosomal clearance of dysfunctional mitochondria (reviewed in Lemasters 2005). This critical step in the fate of a damaged mitochondrion has been only identified in the context of functional characterisation of genes related to autosomal recessive forms of PD (*PARK6* and *PRKN*/*PARK2*), which encode the proteins PINK1, a mitochondrial localised kinase and Parkin, a ubiquitin-E3 ligase (Narendra et al. 2008, Youle and van der Bliek 2012). Indeed, a variety of loss of function mutations of PINK1 (*PARK6*, OMIM: 605,909) and Parkin (*PRKN*/*PARK2*, OMIM: 602,544) have been found in juvenile PD patients (Valente et al. 2004; Kitada et al. 1998). Under physiological conditions, PINK1 is translocated to the inner membrane by the translocase of outer membrane (TOM) and the translocase of inner membrane (TIM) complexes where it is cleaved by the mitochondrial inner membrane rhomboid protease presenilin-associated rhomboid-like protein (PARL) that inactivates it (Baker et al. 2011). Upon depolarisation of mitochondria or ROS accumulation, the mitochondrial import is disrupted, PINK1 cannot be translocated to the mitochondrial inner membrane (MIM) and stays in the mitochondrial outer membrane (MOM) where it accumulates and autophosphorylates, which leads to its activation. Activated PINK1 will then recruit Parkin as a second executioner of mitophagy (Narendra et al. 2010) (Fig. 2). PINK1 phosphorylates Parkin on the S65 of the ubiquitin like domain that leads to an open and active conformation of Parkin. Moreover, PINK1 phosphorylates the ubiquitin itself on the residue S65 (Fiesel et al. 2015; reviewed in Truban et al. 2017). Parkin then ubiquitinates various mitochondrial outer membrane proteins, e.g., Mfn2 (Chen and Dorn 2013). This accumulation of ubiquitinated proteins triggers the recruitment of p62 on the mitochondrial surface (Okatsu et al. 2010). p62 in turn triggers the engulfment of damaged mitochondria in the autophagosome that will lead to its degradation by the autophagy pathway (Okatsu et al. 2010).

The PINK1/parkin pathway is involved as well in the balance between fusion and fission. Indeed, Mfn2 is a substrate of PINK1 and Parkin. Its ubiquitination and phosphorylation lead to its degradation. Which means that upon depolarisation, PINK1 and Parkin accumulate on the MOM in their active form, Mfn2 becomes degraded and mitochondria subsequently fragmented (Chen and Dorn 2013).

Besides PINK1 and Parkin, another PD-linked protein related to juvenile forms of the disease is associated with mitochondrial quality control: DJ-1 (*PARK7*; OMIM: 606,324). Loss of function mutations in the DJ-1 gene have been associated with autosomal recessive early onset PD and are far rarer than PINK1 or Parkin mutations (Bonifati et al. 2003). DJ-1 has been described as a sensor of cellular oxidative stress. When stress occurs, DJ-1 is oxidised and subsequently translocated to the mitochondria (Canet-Aviles et al. 2004). The PD-associated loss of DJ-1 function is related to mitochondrial damage and to an increased vulnerability to complex I inhibition, as has been shown in vivo (Meulener et al. 2006). Moreover, DJ-1 associates with molecular chaperones such as mortalin to protect cells against stress-induced apoptosis (Lev et al. 2008; Li et al. 2005; Yokota et al. 2003). The loss of DJ-1 function has been linked to reduced lysosomal activity and reduced basal autophagy with an accumulation of dysfunctional mitochondria in patient-based cellular models (Krebiehl et al. 2010). This indicates a convergence of DJ-1-related pathogenic pathways with the PINK1/Parkin-mediated mitophagy, as the major cellular degradation pathway for dysfunctional mitochondria.

Recently, another structure has been shown to be implicated in the organellar quality control: the MDVs (Fig. 2c). These vesicles are constituted of a double membrane and are about 70–150 nm in diameter. These vesicles are formed by the mitochondria without the involvement of the fission protein Drp1 (Neuspiel et al. 2008). Two distinct types of MDVs have been described: the ones directed to the peroxisome (Braschi et al. 2010) and the ones directed to the lysosome (Soubannier et al. 2012, b).

Only one protein has been described to travel to the peroxisome from mitochondria, a mitochondrial-anchored protein ligase (MAPL or Mul1) (Braschi et al. 2010). MAPL is responsible for the stabilisation of Drp1 and for the degradation of Mfn2, conjointly increasing mitochondrial fission (Braschi et al. 2009). This process implicates the retromer complex, which is known to carry cargoes from endosomes to the Golgi apparatus. Brashi and collaborators (2010) showed that VPS35 and VPS26, two components of the retromer complex, are recruited to MDVs and bind to MAPL in HEK293T. This process may be important for the regulation of the mitochondrial dynamic, to make sure there is a balance between fission and fusion events. Interestingly, mutations in *VPS35* were recently identified as causing an autosomal dominant form of PD (*PARK17*; OMIM: 614,203; Zimprich et al. 2011; Vilariño-Güell et al. 2011). Mutations in *VPS35* are very rare and account for 0.2% of sporadic cases (Hernandez et al. 2016) (Fig. 1). Functional studies of the most common D620N mutation in VPS35 describe a fragmentation of the mitochondria in neurons (Tang et al. 2015; Wang et al. 2015). Tang and collaborators (2015) explored whether the fusion machinery was impaired in cells expressing VPS35 D620N and discovered that the MAPL levels were increased and Mfn2 levels were decreased. In healthy cells, it is hypothesised that MAPL is degraded via the MDVs that destabilises Drp1 (Braschi et al. 2010) and stabilises Mfn2 expression (Tang et al. 2015). Mutant D620N VPS35 does not bind to MAPL anymore, which subsequently is not degraded, stabilises Drp1 and degrades Mfn2, leading to an increased fission and fragmentation of mitochondria. Moreover, physiological VPS35 can also directly modulate Drp1 activity by binding to Drp1 (Wang et al. 2015) and mediating its degradation via MDVs, so that mutations in *VPS35* interfere at different levels with mitochondrial dynamics.

The second type of MDVs implicates the PINK1/Parkin pathway (McLelland et al. 2014) (Fig. 2). These vesicles have been shown to carry oxidised proteins from the mitochondria to the lysosome in vitro (Soubannier et al. 2012). Importantly, this process does not involve the autophagic machinery, the proteins are directly degraded within the lysosome (Soubannier et al. 2012). The MDV formation has been hypothesised to be triggered mainly by oxidative stress and ROS production, which will create a local activation of the PINK1/Parkin pathway (Sugiura et al. 2014). Indeed, it is thought that locally oxidised proteins and lipids might inhibit the import of PINK1 and therefore its inactivation. PINK1 will then recruit Parkin that will ubiquitinate local mitochondrial proteins. This local activation of the PINK1/Parkin pathway will lead to the budding of vesicles from mitochondria containing oxidised proteins (Sugiura et al. 2014). This means that MDV formation is an event that takes place before global mitophagy and is triggered by ROS, not global depolarisation of the mitochondria. Timewise, MDV formation is thought to take place within 2–6 h following a mild antimycin-A treatment, whereas mitophagy occurs later, within 12–24 h (McLelland et al. 2014), revealing the gradual response to mitochondrial dysfunction involving different mechanisms (Fig. 2).

The relevance of impaired mitochondrial quality control in the pathogenesis of PD is further corroborated by the recent identification of mutations in the VPS13C gene in the autosomal recessively inherited form of juvenile parkinsonism (*PARK23*; OMIM: 616,840; Lesage et al. 2016). We hypothesise that VPS13C could also have a role in organellar quality control of mitochondria. Under physiological conditions, VPS13C was found to be localised in mitochondrial fractions and relocalises to the cytoplasm after mitochondrial damage in monkey and human cell lines (Lesage et al. 2016). The PD-associated loss of VPS13C caused mitochondrial fragmentation, loss of membrane potential and increase PINK1/Parkin-mediated mitophagy after challenging cells with the mitochondrial uncoupler CCCP (carbonyl cyanide 3-chlorophenylhydrazone) (Lesage et al. 2016). It has also been shown to colocalise with the lysosomal fraction in HeLa cells (Yang et al. 2016).

Within the organellar quality control machinery, another aspect of mitochondrial dynamics is crucial to avoid the spreading of malfunctioning mitochondria or ROS: the intracellular transport of mitochondria. Indeed, mitochondria need to travel in the neurons according to local energy demand. Miro1, a mitochondrial Rho GTPase, is implicated in the trafficking of mitochondria (Fig. 2a). Miro1 associates with kinesin-1 and Milton in a calcium-dependent manner to allow mitochondria to travel along microtubules (Wang and Schwarz 2009, MacAskill et al. 2009). When the calcium concentration is high, Miro1 binds calcium and the transport stops at the place where the mitochondria is the most needed. Nevertheless, in the case where mitochondria is malfunctioning, its transport needs to stop. The PINK1/Parkin pathway is once more involved in this phenomenon (Wang et al. 2011). Parkin has been shown to ubiquitinate Miro1, subsequently leading to its degradation by the proteasome (Weihofen et al. 2009). The arrest followed by degradation of dysfunctional mitochondria via the Miro1/PINK1/Parkin pathway avoids spreading of ROS within the cell but also prevents corruption of healthy mitochondria via fusion. Loss of Miro1 can rescue PINK1-related phenotypes by activation of mitophagy and overexpression of Miro1 in a *Drosophila* model revealed an aberrant mitochondrial aggregation and loss of dopaminergic neurons (Liu et al. 2012). Recently, LRRK2 has been shown to interact with Miro1 by promoting its removal from mitochondria in order to arrest the transport of malfunctioning mitochondria in iPSC-derived neurons (Hsieh et al. 2016). These observations strengthen the role of Miro1 in the pathology of PD and made it an interesting candidate gene for PD (Fig. 1). However, no study has yet been able to define a genetic contribution of variants in the Miro1 gene, *RhoT1*, with PD (Anvret et al. 2012).

These studies indicate that impaired organellar quality control pathways have been well established in neurodegeneration in PD, including an increasing number of genes related to mitochondrial homeostasis that are mutated in familial forms of PD. Nevertheless, more recently, another level of quality control has been implicated into molecular pathways related to PD. This level precedes the above-mentioned and is the molecular quality control.

## Mitochondrial dysfunction affecting molecular quality control

The first level of molecular quality control implicates chaperoning proteins. Indeed, most of the proteins found in the mitochondria have a nuclear origin. In order to enter the mitochondria, these proteins need chaperones to undergo unfolding and refolding inside the mitochondria. Proteins with a mitochondrial target sequence enter by TOM and are furthered by TIM (reviewed in Baker et al. 2011). mtHsp60 is typically recruited to help in the folding process together with mortalin (HSPA9), a member of the Hsp70 family (Wadhwa et al. 2015) (Fig. 3a). Damaged or unfolded proteins from the matrix are degraded by mitochondrial proteases, whereas in the OM they are ubiquitinated and degraded by the ubiquitin–proteasome system (UPS) in the cytosol (Livnat-Levanon and Glickman 2011). Two transmembrane AAA metalloprotease complexes are responsible for the quality control across the MIM. Indeed, as the ETC is localised in the MIM, proteins in the intermembrane space (IMS) are more susceptible to ROS and unfolding (Baker et al. 2011). Omi/HtrA2, a serine protease, is thought to play a role in the degradation of proteins in the IMS (Moisoi et al. 2009) (Fig. 3b). Interestingly, two point mutations leading to amino acid exchanges, A141S and G399S, in Omi/HtrA2 (*PARK13*, OMIM: 610,297) have been found in PD patients from a German cohort (Strauss et al. 2005). The G399S mutation was subsequently found to co-segregate with PD and essential tremor in a large family with multiple affected individuals of Turkish descent, providing further genetic evidence for the pathogenic role of G399S mutant Omi/HtrA2 (Unal Gulsuner et al. 2014). Functional characterisation of both mutations in vitro revealed an association with a reduced protease activity and overexpression of the mutant protein leading to mitochondrial dysfunction in human neuronal cell lines, suggesting a dominant negative effect due to the trimer formation required for physiological Omi/HtrA2 function (Strauss et al. 2005). Indeed, the first transgenic mouse model overexpressing human G399S Omi/HtrA2 displayed mitochondrial defects and neurodegeneration that were in line with a dominant-negative effect in vivo (Casadei et al. 2016). Additionally, the loss of function of Omi/HtrA2 is associated with increased ROS (Moisoi et al. 2009), mitochondrial dysfunction (Strauss et al. 2005) and a progressive movement disorder in mice (Jones et al. 2003; Rathke-Hartlieb et al. 2002). This suggests a link between molecular quality control of mitochondria and PD and supports the notion of mutations in the Omi/HtrA2 gene as rare variants with substantial effect (Fig. 1).

If misfolded or oxidised proteins accumulate, the mtUPR takes over, which results in the upregulation of various genes. The mtUPR includes the upregulation of Drp1, the main mitochondrial fission effector but none of the proteins of the fusion machinery (reviewed in Schulz and Haynes 2015). This highlights the fact that, during stress, mitochondria becomes fragmented. In addition, Mfn1 and Mfn2, the GTPase in charge of OM fusion, are degraded by the UPS (Tanaka et al. 2010) and OPA1 isoforms are processed by OMA1, which results in an increase of the shorter isoforms and a decrease of the fusion rate (Baker et al. 2011).

Interestingly, EIF4G1 (eukaryotic initiation factor 4G) (*PARK18*; OMIM: 614,251) has been proposed as a PD-linked protein by Chartier-Harlin and collaborators (2011). As EIF4G1 is a transcription factor, it may also play a role in the transcription of nuclear-encoded mitochondrial proteins important for the mtUPR. Two main variants have been found, R1205H and A502V, in families with an autosomal dominant inheritance of PD (Chartier-Harlin et al. 2011). These variants are very rare (0.2% of sporadics; Puschmann 2013) (Fig. 1) and thought to perturb the binding of mRNA to ribosomes (Deng et al. 2015). Even though the precise role of these mutations in PD is unknown and the causal link of genetic variants with PD is still debated (Huttenlocher et al. 2015), EIF4G1 variants have been linked to mitochondrial dysfunction. Indeed, upon oxidative stress, mitochondria of cells overexpressing the EIF4G1 PD-linked variants, have difficulties in rapidly and dynamically responding to stress compared to wild-type EIF4G1 overexpressing cells (Chartier-Harlin et al. 2011).

mtUPR promote an increase of mitochondrial chaperones (mtHsp60, mortalin, TRAP1) and proteases transcription to support the recovery of the mitochondria (Zhao et al. 2002) (Fig. 3b). Also, transcription of glycolysis genes and lactate dehydrogenase are increased, which show a switch to oxidative glycolysis during mitochondrial stress (Mouchiroud et al. 2013).

However, two additional mitochondrial chaperones have been linked to PD pathogenesis based on rare disease-associated variants: mortalin and TRAP1 (Fig. 1). Mortalin, encoded by *HSPA9*, has been thought to be a candidate for causing PD, due to selective downregulation in the substantia nigra of patients compared to controls, strengthening the relevance of fine-tuned proteostasis for the correct function of mitochondria (Jin et al. 2006). Subsequently, three rare genetic variants were described as associated with PD in a Spanish and German cohort (De Mena et al. 2009; Burbulla et al. 2010). Functional characterisation of these variants supported a role as susceptibility factors, as all variants caused increased intramitochondrial ROS levels and reduced MMP in different human cell lines (Burbulla et al. 2010).

Interestingly, the impaired intramitochondrial molecular quality control due to reduced mortalin function led to an increased autophagic clearance of damaged mitochondria and subsequently to a reduced mitochondrial mass in human cells in vitro and ex vivo (Burbulla et al. 2014). Increased mitophagy via Parkin or PINK1 overexpression rescued the loss of mortalin-associated mitochondrial phenotypes and required an intact autophagic pathway. This convergence was in line with the phenotypes observed in fly models of PD in vivo, as partial loss of mortalin in Drosophila recapitulated impaired motor phenotypes observed in loss of Parkin and loss of PINK1 function (Zhu et al. 2013). Here, dopaminergic neurons were more susceptible to cell death induced by reduced mortalin function than other neuronal populations or non-neuronal cells. Thus, the partial loss of mortalin function provided a first direct link between impaired molecular quality control related to mtUPR and PD pathogenesis.

TRAP1 is part of established PD signalling pathways, as it has been shown to be phosphorylated and activated by PINK1 (Pridgeon et al. 2007). A rare mutation of TRAP1 causing loss of function was identified in a typical late-onset PD patient (Fitzgerald et al. 2017). Functional analysis in patient-derived cells suggests that loss of TRAP1 results in significant loss of mitochondrial membrane potential and sensitivity to late-stage quality control such as organellar removal and apoptosis. Moreover, overexpression of TRAP1 in *Drosophila* is able to compensate for PINK1 loss of function (Zhang et al. 2013) and mutant A53T alpha-synuclein-mediated mitochondrial toxicity (Butler et al. 2012). Recently, TRAP1 was described to interact with Omi/HtrA2 and rescued mitochondrial phenotypes associated with a loss of Omi/HtrA2 function) suggesting a signalling pathway downstream of PINK1 and Omi/HtrA2 (Fitzgerald et al. 2017).

In summary, there is increasing evidence strengthening the link between an impaired molecular quality control and molecular pathways related to neurodegeneration in PD.

## Mitochondrial dysfunction affecting cellular integrity

In cases of sustained mitochondrial dysfunction, apoptosis of the cell is the last solution to avoid general damage to the organism. Apoptosis is a physiological event occurring during development and arising when molecular and organellar quality controls are overwhelmed. Two distinct pathways have been described, the extrinsic and the intrinsic pathways, with the latter involving a mitochondrial signalling pathway. Here, upon cellular stress, Bax and Bak are translocated in the mitochondrial OM where these proteins colocalise with mitochondrial fission sites (Karbowski et al. 2002). In order to trigger apoptosis, these pro-apoptotic proteins need to outbalance anti-apoptotic proteins like Bcl-2 and Bcl-xL (Ghavami et al. 2014). Bax and Bak then contribute to the release of cytochrome c through the permeability transition pore (Fig. 3c). Cytochrome c will subsequently form a complex with pro-caspase-9, which activates caspase-9, the initiator caspase. Caspase-9 will in turn promote the activation of caspase-3, the executioner caspase and lead to the activation of the apoptosis pathway.

Interestingly, mitochondria-mediated apoptosis can be regulated by CHCHD2, a gene identified as responsible for an autosomal dominant of typical PD (*PARK22*; OMIM: 616,710; Funayama et al. 2015). It has been shown that CHCHD2 binds to Bcl-xL and thereby supports the anti-apoptotic interaction between Bcl-xL and Bax (Liu et al. 2015). Under stress conditions, CHCHD2 induces a relocalisation of Bax to mitochondrial membrane followed by an opening of the mitochondrial permeability transition pore (Fig. 3c). Moreover, CHCHD2 has been shown to bind to the ECL component cytochrome c oxidase (Aras et al. 2015). When the levels of CHCHD2 were decreased, mitochondrial impairment occurs, as defined by decreased MMP, increased ROS levels and mitochondrial fragmentation (Aras et al. 2015). These observations indicate a role of CHCHD2 in balancing both apoptosis and mitochondrial function and reinforce the important interplay between these processes to insure cellular homeostasis.

VPS35 also has an anti-apoptotic role via its association with Lamp2a and with the Parkin substrate, aminoacyl-tRNA synthetase complex interacting multifunctional protein-2 (AIMP2) (Yun et al. 2017). For VPS35 harbouring the PD-associated D620N mutation, this association was disturbed and led to an increased level of non-degraded AIMP2, which translocates to nucleus and activates PARP1 leading to cell death (Yun et al. 2017; Lee et al. 2013).

Moreover, Mortalin and TRAP1, together with mtHsp60, have been shown to be implicated in the control of cytochrome c release (Ghosh et al. 2010; Qu et al. 2012) (Fig. 3c). Depending on the subcellular localisation, Omi/HtrA2 can also have a pro-apoptotic effect after release from the mitochondria. Indeed, in the cytoplasm, it has been shown to bind to inhibitor of apoptosis proteins (IAPs) and therefore promote the activation of caspases and apoptosis (Verhagen et al. 2002) (Fig. 3c).

As described above, several genes implicated in monogenic forms of PD are playing an important role not only at one but at different levels of mitochondrial quality control. These models can serve as prototypes to identify signalling pathways related to impaired mitochondrial functions. Even if these familial forms represent less than 10% of all PD cases, the lessons learned on the role of mitochondrial integrity in PD may be also relevant for the typical sporadic PD cases.

## Genetic risk factors linked to impaired mitochondrial function in sporadic PD

More and more, the genetic background of patients with sporadic forms of PD appears to be of importance. Besides the rare genetic variants with a high effect size defining monogenic forms of PD, there is an increasing number of genetic risk factors with a smaller effect that may also contribute to neurodegeneration. Among these, susceptibility factors or mutations with reduced penetrance, e.g., mutations in *GBA*, *LRRK2* and some *SNCA* variants, are blurring the frontier between familial and idiopathic PD (Fig. 1). Some of these mutations are leading to an impairment of neuronal integrity, notably caused by mitochondrial dysfunction.

Autosomal dominantly inherited mutations in the *SNCA* gene encoding alpha-synuclein (*PARK1/PARK4*; OMIM: 168,601, 605,543) were the first identified genetic cause for PD (Polymeropoulos et al. 1997; Krüger et al. 1998). Subsequently, additional rare mutations in the *SNCA* gene were discovered, including point mutations, duplications or triplications. The penetrance of *SNCA* point mutations is indicating a strong effect in terms of pathogenicity. Interestingly, a potential role of the *SNCA* gene also in the sporadic form of the disease was early suggested by the presence of alpha-synuclein in Lewy bodies, the pathological hallmark in brains of PD patients. Common genetic variants in *SNCA* gene were subsequently identified without strong causal effect but rather appear as risk factors for sporadic PD. Among them, the complex polymorphic microsatellite repeat, NACP-Rep1, located 10 kb upstream of the transcription start site of *SNCA* has been early associated with sporadic PD (Krüger et al. 1999; Maraganore et al. 2006; Kay et al. 2008). The presence of the risk allele, Rep1-261 bp, of the dinucleotide repeat polymorphism in the promoter of *SNCA* leads to an increase of alpha-synuclein expression in vitro and in vivo (Chiba-Falek and Nussbaum 2001; Cronin et al. 2009), leading to increased protein levels in blood of PD patients carrier of this variant compared to the protective genotype (Fuchs et al. 2007). Indeed, in analogy to the disease-causing effect of the gene duplication or triplication, a critical dose-effect with a slight but significant increase of alpha-synuclein expression due to the polymorphism could suffice to cause the late onset sporadic form of PD, as opposed to the severe early onset form caused by an *SNCA* triplication. Interestingly, increased levels of alpha-synuclein have been shown to induce mitochondrial fragmentation by direct interaction (Kamp et al. 2010; Nakamura et al. 2011) but also to cause impaired energy balance due to decrease respiration rates and ATP production (Flierl et al. 2014; Sarafian et al. 2013). Moreover, an increase of alpha-synuclein has been correlated with an increased Ca2+ transfer from the ER to the mitochondria, which may contribute to oxidative stress (Calì et al. 2012) (Fig. 3c). Indeed, increased cellular stress and ROS levels have been observed in stem cell-derived neuronal precursor cells of a PD patient carrying a *SNCA* triplication (Flierl et al. 2014). Pukaß and colleagues showed that the application of mitochondrial stressors induced a decrease of autophagic clearance of alpha-synuclein, which causes alpha-synuclein accumulation (Pukaß et al. 2015). These studies underscore a relationship between alpha-synuclein and mitochondrial homeostasis that may trigger cellular quality control via apoptosis. Moreover, several SNPs located in the 3′ end of SNCA have been found to be associated with PD (Simón-Sánchez et al. 2009; Mueller et al. 2005). Interestingly, carriers of the ‘G’ allele of SNP rs356219 display increased levels of alpha-synuclein in the blood (Mata et al. 2011) but decreased SNCA mRNA expression in brain, particularly in SN (Fuchs et al. 2007; Linnertz et al. 2009). This indicates a potential tissue-specific modulatory effect of different alleles of rs356219 on the SNCA mRNA levels. Moreover, a pilot study has shown that PD patients carrying the ‘G’ allele have a more favourable treatment response to deep brain stimulation therapy (Weiss et al. 2016). Indeed, rs356219 was associated with a subtype of PD without cognitive impairment, which may indicate a lower burden of alpha-synuclein aggregation in different brain regions related to preserved basal ganglia circuits as a basis for optimal DBS effect (Guella et al. 2016; Weiss et al. 2016). The mechanism underlying the *SNCA* SNP rs356219 needs further studies to fully understand its pathogenicity but it appears here, in line with the critical dose hypothesis, that different levels of alpha-synuclein in specific brain regions may contribute not only to the risk to develop sporadic PD but also influence the therapeutic outcomes.

Heterozygous mutations in the *GBA* gene, encoding the lysosomal enzyme glucocerebrosidase (GCase), are the most common risk factor for sporadic PD with 3–7% of all PD patients harbouring a mutation in this gene (Sidransky et al. 2009; Lesage et al. 2011). *GBA* mutation carriers have an up to 20-fold increased lifetime risk of developing PD (Schapira 2015). Nevertheless, the penetrance of 20% at age 70 and 30% at age 80 excludes *GBA* variants as a clear monogenic form of PD (Anheim et al. 2012). Physiologically, GCase is responsible for the hydrolysis of glucosylceramide into glucose and ceramide that will be integrated into membranes. Moreover, GCase participates to the lysosomal degradation of alpha-synuclein that, when accumulating, can in turn impair GCase trafficking (Mazzulli et al. 2011). Homozygous mutations in the *GBA* gene have been extensively studied in models of Gaucher’s disease (GD) and are known to lead to autophagolysosomal dysfunction, accumulation of glucosylceramide and lipid metabolism impairment (Panicker et al. 2012; Magalhaes et al. 2016). *GBA* loss of function has been shown to induce mitochondrial impairment as revealed by a decreased MMP and reduced ATP production in different in vitro and in vivo models (Cleeter et al. 2013; Osellame et al. 2013; de la Mata et al. 2015). Moreover an increase of oxidative stress and fragmentation of mitochondria have been observed under inhibition of GCase and in a mouse model of GD (Cleeter et al. 2013; Osellame et al. 2013). The direct link between *GBA* and mitochondrial dysfunction may be related to quality control from its role of hydrolase. First, lack of GCase appears to decrease macro-autophagic flux (Osellame et al. 2013; Schöndorf et al. 2014) as revealed by an impaired lysosomal function (Fig. 2). This defect in organellar quality control leads to a decrease of mitophagy and causes accumulation of dysfunctional mitochondria unable to ensure their role in the cell. Autophagic impairment is particularly detrimental for neuronal cells, which are postmitotic and therefore cannot dilute their damaged organelles or unfolded proteins by cell division. Moreover, an impaired GCase function unbalances alpha-synuclein degradation and may overload the lysosome with subsequent accumulation of alpha-synuclein (Mazzulli et al. 2011; Schöndorf et al. 2014; Yang et al. 2017). As mentioned previously, alpha-synuclein accumulation can independently lead to mitochondrial dysfunction and apoptosis (Fig. 3c). Proteostatic burden of aggregated alpha-synuclein can challenge an already mildly impaired lysosomal function, which may synergistically lead to cellular dysfunction. Finally, Schöndorf and colleagues observed an increased level of cytosolic Ca2+ in neurons derived from iPSC of *GBA* mutation carriers (Schöndorf et al. 2014). Accumulation of misfolded mutant GCase into the ER leads to dysfunction of the UPR (Kurzawa-Akanbi et al. 2012; Fernandes et al. 2016) and can perturb ER related Ca2+ homeostasis (Kilpatrick et al. 2016). The fact that *GBA* mutations appear as a risk factor for PD in the heterozygous state shows that one healthy allele may be enough to ensure normal cellular function for a certain time; however, during ageing, mutant cells cannot cope anymore with lysosomal and mitochondrial dysfunctions associated to alpha-synuclein accumulation. To better understand this transition, more studies are needed on the specific link between *GBA* and PD as most of the existing studies focus on GD models or chemically induced by GCase inhibition.

Mutations in the *LRRK2* gene (*PARK8*; OMIM: 607,060) are the most frequent cause of autosomal dominant PD (Lesage and Brice 2009) but, interestingly, some population-specific mutations in this gene with reduced penetrance appear as well in sporadic cases (Lesage and Brice 2012). Seven LRRK2 mutations have a proven pathogenicity (Healy et al. 2008; Lesage and Brice 2009). Among them, the G2019S mutation has a frequency worldwide of 1–2% in sporadic cases (with up to 30% in northern Africa; Benamer and De Silva 2010) and 4–5% in hereditary PD (Healy et al. 2008; Nichols et al. 2005; Gilks et al. 2005) with a variable penetrance (G2019S: 28% at 59, 51% at 69 and 74% at 79 years; Healy et al. 2008). This observation already highlights the dual role of *LRRK2* as a causal gene for familial PD and as a risk factor for sporadic PD. The G2385R and R1628P variants of *LRRK2* are particularly frequent in the Asian population, where they cause a 2- to 3-fold increased risk for PD (Wu et al. 2013; Gopalai et al. 2014). With a frequency of 3–5% in the Asian PD population, these two variants can be considered as the most common variants for developing PD in East Asia (Pulkes et al. 2014).

The G2385R mutation is localised in a protein–protein interaction domain (WD40) and may disturb interaction with binding partners or diminish dimerization of LRRK2 (Mata et al. 2006). Contrary to the G2019S mutant, the kinase activity of LRRK2 G2385R is decreased and its GTPase activity is increased (Ho et al. 2016; Rudenko et al. 2012). Under conditions of oxidative stress, the G2385R variant leads to a higher rate of apoptosis (Tan et al. 2007). Also, neurons from transgenic *Drosophila* for G2385R were more susceptible towards mitochondrial toxins (Ng et al. 2009). From these studies, it appears that the G2385R variant induces cellular dysfunction only in the presence of additional environmental stress. Rudenko and colleagues detected an increased binding of LRRK2 G2385R to the chaperone Hsp90, which would imply a refolding of the protein (Rudenko et al. 2012). They further showed that LRRK2 G2385R protein levels are decreased due to its higher affinity for CHIP, which induced proteasomal degradation of the protein (Rudenko et al. 2017). Under physiological conditions, the balance between refolding of proteins by chaperones and proteasomal degradation is tightly regulated. Cellular stress like ageing, unbalanced homeostasis or increased ROS levels can turn in favour of degradation (Pratt et al. 2010).

The R1628P mutation is localised in the COR (C-terminus of ROC) domain of LRRK2. The amino acid exchange may lead to conformational changes and could affect dynamic interaction among LRRK2 domains (Ross et al. 2008). Shu and colleagues showed that R1628P does not directly alter kinase activity of LRRK2 but increases its affinity for Cdk5 by allowing phosphorylation of the preceding serine that will activate the kinase function (Shu et al. 2016). Cdk5 is activated by oxidative stress via activation of the Ca2+ dependent protease calpain (Strocchi et al. 2003; Dhavan and Tsai 2001). Neurons carrying the R1628P variant have a higher sensitivity to MPTP (Shu et al. 2016). Indeed, disturbance of mitochondria by MPTP leads to production of oxidative stress that would activate Cdk5, which in turn leads to hyperphosphorylation of LRRK2 R1628P variant and increases its kinase activity. Of note, the mechanism could be dependent on a dose effect, as patients homozygous for R1628P display a stronger phenotype (Lu et al. 2008). Cellular dysfunction caused by R1628P variant need further studies but the previous data are showing that unbalanced mitochondrial homeostasis could be the trigger of increased kinase activity that would lead to typical LRRK2 dysfunction as aberrant vesicular trafficking and protein synthesis (Martin et al. 2014). Reprogramming of peripheral blood mononuclear cells from patients with PD harbouring heterozygous R1628P mutation into iPSC (Ma et al. 2017) will provide a better model to decipher the R1628P effect.

Together, these studies show that more frequent genetic variants with weak to moderate effects can substantially contribute to the risk to develop sporadic PD and implicate mitochondrial impairments to different extents and at different levels of mitochondrial quality control.

## Conclusion

There is increasing evidence that PD is a heterogenous disorder involving different genes and different molecular pathways, all converging to a characteristic (but not exclusive) degeneration of dopaminergic neurons in the substantia nigra. Therefore, novel approaches in defining access to causative neuroprotective therapies have to account for this heterogeneity and need to define criteria for stratification of sporadic PD into different subgroups. Among the different signalling pathways described in PD, mechanisms implicating impaired mitochondrial homeostasis get more and more into focus. Also, an increasing number of monogenic forms of PD have been identified that have helped to dissect the different instances of mitochondrial quality control involved in the neurodegenerative process. Here, the PINK1/Parkin-mediated pathway for organellar quality control is representing an important building block in the understanding of the molecular underpinnings of PD. Based on genetically defined models of PD, new approaches can now be taken to develop neuroprotective treatment strategies. Using patient-derived fibroblasts from Parkin mutation carriers, the first successful compound screening campaign was recently published that allowed to define bile acid derivatives as potential candidates for reverting PD-associated mitochondrial phenotypes like energy deficiency and altered mitochondrial morphology (Mortiboys et al. 2015). Interestingly, the same class of compounds was also effective in patient-based models with another monogenic form of PD, due to mutations in the *LRRK2* gene (Mortiboys et al. 2015). This may indicate that strategies defining neuroprotective therapies using monogenic models related to primary mitochondrial dysfunction may also be effective in forms of PD that involve secondary mitochondrial damage.

However, even all monogenic forms of PD taken together only contribute to a minority of all patients and therefore strategies to define the mitochondrial subtypes within the most common sporadic form of PD need to be developed. Besides rare genetic variants that interfere with proper mitochondrial function and confer substantial risk to develop typical PD, also more common variants, e.g., in the *GBA* or *LRRK2* genes were defined that may interfere with mitochondrial homeostasis. This adds to the complex genetic architecture of PD and led to the concept of a certain genetic burden within a pathway, e.g., related to mitochondrial quality control and that confers risk to the more common sporadic form of PD. Indeed for some heterozygous carriers of mutations in the *PARK2* or *PINK1* gene an increased risk to develop sporadic PD has been described (Hilker et al. 2001; Oliveira et al. 2003; Foroud et al. 2003) and also digenic cases of PD revealing an interplay of mutations in different genes related to the same pathology have been reported (Funayama et al. 2008). Recent studies using functional prioritisation of candidate genes derived from next generation sequencing strategies further extended the concept of genetic burden that may implicate several ‘hits’ in one individual defined by variants in different genes, that per se only have a minor effect but may add up to a relevant effect on mitochondrial function (Jansen et al. 2017). Therefore a similar approach to what has been outlined here can be justified for other disease-related pathways, e.g., involving impaired endosomal-lysosomal function. Indeed there is increasing evidence of monogenic causes of PD (*PARK19*, *PARK20* or *PARK21*) that are directly involved in the shuttling of vesicles and maturation into lysosomes for degradation of not only mitochondria but also other organelles and protein aggregates (Edvardson et al. 2012; Köroĝlu et al. 2013; Krebs et al. 2013; Quadri et al. 2013; Vilariño-Güell et al. 2014). Using mechanism-based stratification more precise therapeutic interventions will be developed that account for the interindividual differences and the heterogeneity of PD (Krüger et al. 2017).
